# Study of the ordered assembly morphologies of diblock copolymers on the same substrate

**DOI:** 10.1039/d2ra04803e

**Published:** 2022-10-05

**Authors:** Baolin Zhang, Lingkuan Meng, Zili Li

**Affiliations:** School of Information Science and Technology, Fudan University Shanghai 200433 China lizili@fudan.edu.cn; Beijing Institute of Carbon-based Integrated Circuit Yiyuan Cultural and Creative Industry Park, 80 Xingshikou Road, Haidian District Beijing 100089 China

## Abstract

With the development of frontier technology in emerging semiconductor processes, self-assembling (SA) and directed self-assembly (DSA) of block copolymers (BCPs) have attracted great attention from scientific researchers and become promising candidates for advanced photolithography. Using an optimal coating and baking process, highly ordered assembly morphologies (*e.g.*, cylinder and lamella) of two BCPs in thin films were obtained without an additional topcoat material layer. Moreover, the whole experimental study also provides an optimal process for integrating the two BCPs into the same topographic guiding pattern substrate fabricated by electron beam lithography (EBL) to achieve specific self-assembly. This topographic guiding substrate achieves not only lamellar micro-domains aligned perpendicular to the sidewalls of trench edges but also cylindrical micro-domains (PMMA phase in a PS matrix) aligned parallel to trench edges respectively, which provides insights and valuable information for further applications in lithography and electronic devices.

## Abstract 2

With the development of frontier technology in emerging semiconductor processes, directed self-assembly (DSA) of block copolymer has attracted great attention from scientific researchers and become promising candidate for advanced photolithography.

## Graphical abstract

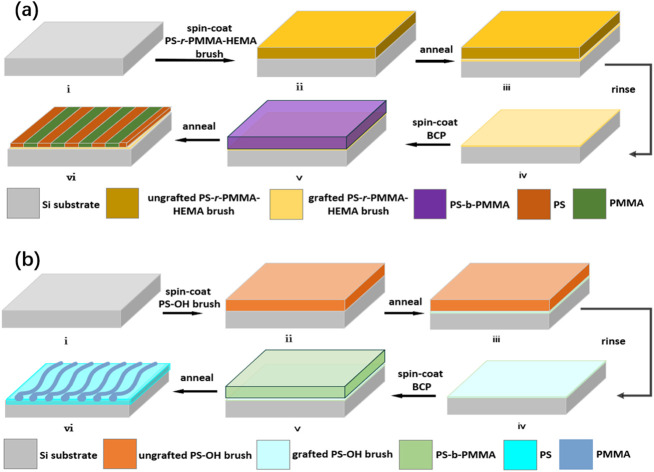

## 1. Introduction

Conventional top-down lithography for semiconductor devices meets the expected technology nodes using immersion ultraviolet lithography for advanced photolithography. Extreme ultraviolet (EUV) technology has driven the extension and advancement of the sub-7 nm node or even smaller sizes. Still, it turns out that the cost of this complex technology is particularly huge.^1^ Hence, versatile, compatible strategies, and low-budget routes with bottom-up technologies are the research directions for next-generation advanced micro–nano electronic devices. In recent years, block copolymer self-assembly has become a promising technique for fabricating advanced semiconductor devices such as ultrafiltration membranes,^2^ memory devices,^3^ metallic nanoparticles, arrays of quantum dots,^4,5^ bit-patterned media,^6^ and nanowire-based transistors^7,8^ because BCPs have the intrinsic ability to form highly ordered periodic nanostructures. The extensive development of BCP microphase separation of lamellar and cylindrical morphologies has opened up a novel approach for next-generation nano-lithography.^9–11^ Self-assembly refers to the spontaneous formation of well-defined arrangements of molecules, or in other words, the BCP forms a long-range ordered morphology to minimize the system free energy.^12^ The minimum size that can be achieved depends on the molecular weight of BCPs, in which the size of the nanopattern that can be achieved is about 5–100 nm.^13,14^ The critical aspect in the DSA lithography is block copolymers, which are composed of two or more covalently bonded polymers with different properties that can self-assemble into periodic nanostructures with different morphologies. Indeed, relevant guiding patterns are usually desired to induce BCP self-assembly into large-scale well-registered nanopatterns with ordered orientation. The guiding patterns can be created by graphoepitaxy or chemoepitaxy.^15,16^ The pitch size of BCP nanostructure is determined by the copolymer's degree of polymerization (*N*) and Flory–Huggins parameter (*χ*) (*L*_0_ ≈ *χ*^1/6^*N*^2/3^).^17^ The development and advancement of DSA are positively related to the intrinsic properties of materials, in which the main line and spacing of BCP pitch size dominate the resolution of DSA patterns and related applications. The block copolymer self-assembly process has many advantages such as the fast realization of nanopatterns with high precision and low cost, whereas conventional photolithography cannot easily be achieved. Simultaneously, the combination of bottom-up and top-down approaches to nanopatterning has also attracted more attention from researchers.^18,19^ The common diblock copolymer polystyrene-*b*-polymethylmethacrylate (denoted PS-*b*-PMMA) is synthesized from PMMA and PS homopolymers. It can form lamellar, cylindrical, spherical, and gyroid microphase separation by changing the volume ratio of BCP components.^20–22^ Highly ordered and unified line/spacing or hole arrays have been achieved by guiding these morphologies. They can also be used as etching masks for the fabrication of various nanostructures. The lamellar structure forms a nano line or array pattern *via* selective etching, which can be further used as a mask to achieve the pattern transfer on different substrates. Ordered assembly morphologies have been achieved to fabricate fin or gate arrays in high-performance and complex FinFET devices.^23–25^ Certainly, the vertical cylinder structures can be used to make contact holes or channel holes in CMOS devices,^26^ while the parallel cylinder structures can also be used as nanowire sensor applications. Relevant research has been manipulated to balance the surface energy between the blocks and the substrate to achieve the vertical orientation of BCPs. In general, the neutral layer is a random copolymer that shows a similar character to the two blocks of the diblock copolymer. Different BCP materials need corresponding neutral materials (or material that tends to interact with a block) and appropriate film thicknesses to control surface energies, and then accurately control the microphase separation of SA and DSA structures.^27,28^ However, there are few studies on the SA (or DSA) morphologies of two different types of diblock copolymers on the same silicon substrate (or silicon groove substrate), respectively. It should be noted that the combined innovative experimental scheme is to maximize the utilization of existing materials from the perspective of timeliness and cost, which is of great significance and value for the versatility and multifunction of future applications in semiconductors.

Herein, the highly ordered assembly morphologies of SA and DSA with lamellar and cylindrical BCPs on the same substrate were studied under the same process condition. We carried out relevant experimental research, including the preparation of the two BCP thin films, the orientation of microdomains of SA on the Si substrate, the orientation of microdomains of DSA in the Si substrate groove, a theoretical analysis, and the intention of our experimental research. From the point of view of practicality and cost, Si substrate grooves of different widths with the same depth have also been successfully prepared by EBL for the study of the two types of BCPs. By tuning the surface energy of both sidewalls and bottom surface to be nonselective, the lamellar alignment perpendicular to the sidewalls has been achieved and the line/spacing width of nanowires has almost no change. In addition, a cylindrical block copolymer on the same groove-induced Si substrate has been employed to achieve nanowire arrays that align parallel to the trench sidewall. Moreover, for the cylinder phase, the change in line/spacing width of nanowires from the edge of the groove to the center has certain regularity. Most importantly, the block copolymers succeeded in achieving highly ordered DSA nanopatterns under optimal process conditions. We also carried out the corresponding mechanism analysis, which provides valuable information on DSA lithography for the future fabrication and development of semiconductor devices.

## 2. Experimental methods

### 2.1 Materials

An approximately symmetric diblock copolymer material, PS-*b*-PMMA (denoted PS-*b*-PMMA1), PDI = 1.08, *M*_n_ = 31-*b*-33 kg mol^−1^, and a random copolymer brush (denoted PS-*r*-PMMA–HEMA), *M*_n_ = 7 kg mol^−1^, *M*_w_ = 10.4 kg mol^−1^, *M*_w_/*M*_n_ = 1.48, were used. The other asymmetric diblock copolymer material, PS-*b*-PMMA (denoted PS-*b*-PMMA2), PDI = 1.09, *M*_n_ = 46-*b*-21 kg mol^−1^, and a monohydroxy-terminated polystyrene (denoted PS–OH), *M*_n_ = 6 kg mol^−1^, *M*_w_ = 6.4 kg mol^−1^, *M*_w_/*M*_n_ = 1.05, were all purchased from Polymer Source, Inc. Toluene, isopropanol and acetone were all purchased from Sigma-Aldrich. Silicon wafers, 4 inches, P-type (100), were purchased from WRS Materials. Electron beam photoresist PMMA950 and developing solution methyl isobutyl ketone (MIBK) : isopropanol (IPA) (1 : 3) were purchased from Suzhou Yancai Weina Technology Co., Ltd. The grade of solvents used in the experiments was analytical reagent (AR). All other chemicals were used as received.

### 2.2 Spin coating process of PS-*r*-PMMA–HEMA and PS–OH brush layers on a silicon substrate

Wafers of 4 inch Si (100) with approximately 1.4 nm-thick silicon oxide layers were cut into two-centimeter squares, which were first immersed in acetone solution at 33 °C and sonicated with 40 kHz and 50% ultrasonic power for 6 min, and then immersed in isopropanol (IPA) at 33 °C and sonicated with 40 kHz and 50% ultrasonic power for 6 min. Finally, the wafers were rinsed and dried under a nitrogen flow to remove relevant pollutants. The random copolymer PS-*r*-PMMA–HEMA brush layer was obtained by spin-coating a 1 wt% toluene solution onto the cleaned silicon substrate. The other monohydroxy-terminated polystyrene (PS–OH) brush layer with approximately 30 nm thickness was obtained by spin-coating 0.5 wt% PS–OH toluene solution onto the cleaned silicon substrate. The samples were annealed at 240 °C in a nitrogen atmosphere for 10 minutes to form a grafted layer. Then, the samples were placed in a toluene solvent for ultrasonic cleaning, the ungrafted polymer brush was removed, and the samples were dried in nitrogen. The monohydroxy-terminated polystyrene (PS–OH) brush layer was fabricated by the same process. Finally, the PS-*r*-PMMA–HEMA brush layer with an approximate thickness of 3 nm and the PS–OH brush layer with an approximate thickness of 4 nm were formed on Si substrates, respectively.

### 2.3 Preparation of BCP films on the modified Si substrate

Two kinds of PS-*b*-PMMA solutions (1 wt%) were both spin-coated onto the PS-*r*-PMMA–HEMA-modified and PS–OH-modified Si substrates with different rotation speeds for 60 s to deposit BCP films with different thicknesses, respectively. These silicon substrates were then annealed at 240 °C in a nitrogen atmosphere for 4 minutes to generate microphase separation morphology. The highly ordered self-assembly lamellar pattern on the Si substrate is realized with symmetric BCPs, and the highly ordered morphology of the PMMA cylinders in the PS matrix is also realized on the PS–OH modified Si substrate with asymmetric BCPs under the same process conditions.

### 2.4 Fabrication of directional template features

The DSA morphology of BCP thin films was also examined using Si trench templates 55 nm deep to induce self-assembly. We perform the DSA process on the silicon template containing several different widths of the same depth. The trenches were patterned by EBL with an optimal e-beam exposure dose of 710 μC cm^−2^ using a state-of-the-art beam writer (denoted as JBX6300, JEOL ltd) with a beam spot size of about 10 nm at 100 keV. Post-exposure bake (PEB) was done on a hot plate at 150 °C for 80 s, and then the sample was immediately developed in MIBK : IPA (1 : 3) at 23 °C for 60 s, which was then rinsed with deionized water (DIW) and dried in N_2_ to produce several required different photoresist grooves widths. After that, the remaining photoresist is used as an etching mask to get a certain groove depth by fluorine-containing etching gas, removed by *N*-methyl-2-pyrrolidone solution immersion, rinsed with IPA and then with DIW, and finally dried in N_2_ to produce several required different groove widths.

### 2.5 BCP films for directed self-assembly

Two different types of 1 wt% PS-*b*-PMMA toluene solution were spin-coated onto Si groove templates with several required different widths of the same depth, respectively. These groove substrates were then annealed at 240 °C in a nitrogen atmosphere for 4 minutes to induce directed self-assembly of the BCP. The two experiments were successively carried out on the same groove substrate template made in advance, and the two experiments did not affect each other with surface modification by different brushes.

### 2.6 Characterization

A scanning electron microscope (SEM) of ZEISS SIGMA HD was used to take images at an accelerating voltage of 5 kV. In order to obtain the original morphology of BCP films, oxygen plasma etching or gold metal sputtering were not applied to enhance its contrast. An atomic force microscope (AFM, Park model) was used to measure the guide groove depth of the silicon substrate. Under photoresist PMMA950 as the etching mask, the silicon template containing several different widths of the same depth trenches was successfully implemented by fluoride etching gas for reactive ion etching (RIE, Oxford 100). The corresponding width of the BCP line in SEM images was processed after multiple measurements.

## 3. Results and discussions

**Fig. 1:**
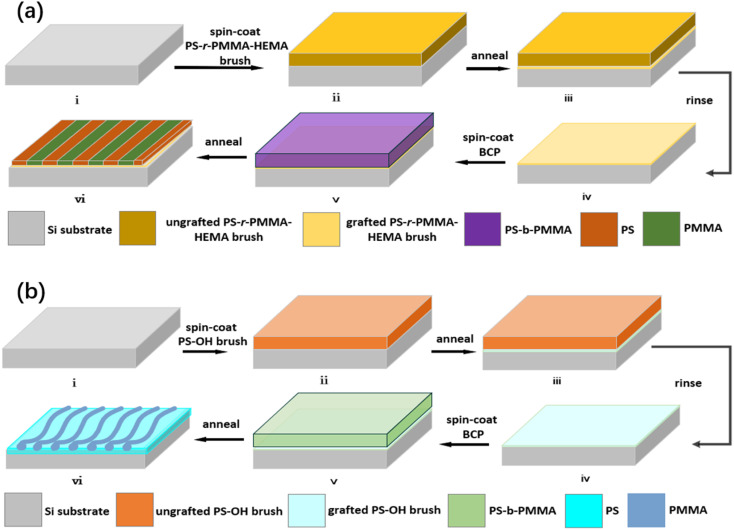
(a). Schematic diagram of the self-assembling process flow of PS-*b*-PMMA1 lamellar domains. (i) Cleaned silicon substrate. (ii) PS-*r*-PMMA–HEMA brush film was deposited on the Si substrate. (iii) Removal of ungrafted PS-*r*-PMMA–HEMA brush. (iv) Graft of the PS-*r*-PMMA–HEMA brush. (v) PS-*b*-PMMA1 deposited on the Si substrate treated with the PS-*r*-PMMA–HEMA brush. (vi) PS-*b*-PMMA1 perpendicular microphase separation after thermal annealing. (b). Schematic diagram of the self-assembling process flow of the PS-*b*-PMMA2 cylindrical domains. (i) Cleaned silicon substrate. (ii) PS–OH film was deposited on the Si substrate. (iii) Removal of ungrafted PS–OH brush. (iv) Graft of the PS–OH brush. (v) BCP was deposited on the substrate treated by the PS–OH brush. (vi) PS-*b*-PMMA2 microphase separation after thermal annealing.

The SA and DSA of BCP thin films are particularly promising lithography to achieve high-resolution patterning for future semiconductor devices. We use post-optimized vacuum thermal annealing accompanied by nitrogen purging, which is a very effective process method because of its excellent feasibility and compatibility with the CMOS semiconductor fabrication flow. Both multitudinous production and low defectivity are extremely significant considerations for using the vacuum thermal annealing process. Certainly, we adopted the optimal BCP self-assembly process in this part, and the schematic diagram of the self-assembly of the BCP solutions spun on the surface of the same Si substrate is shown in Fig. 1. As shown in Fig. 1(a), first, the pre-prepared Si substrate was cleaned. Second, the Si substrate surface was neutralized by grafting PS-*r*-PMMA–HEMA under thermal annealing at 240 °C for 10 minutes, and the ungrafted polymer was washed with toluene to obtain a neutral brush layer, which can uniformly wet both PMMA and PS equally, and it is helpful to achieve perpendicular lamellar nanostructures after post-optimized vacuum thermal annealing. Finally, PS-*b*-PMMA1 was spin-coated onto the neutralized Si substrate and then perpendicular lamellar microphase separation was achieved by vacuum thermal annealing at 240 °C for 4 minutes. To improve the productivity and reduce the defect rate of the process flow, multiple groups of experiments with different thicknesses, annealing temperatures, and durations were finally found under the optimal process condition for SA and the subsequent DSA of the PS-*b*-PMMA1.

Fig. 1(b) shows the process flow of the self-assembly of cylindrical PS-*b*-PMMA2. The PS–OH brush solution was spin-coated and annealed for crafting the PS layer onto the cleaned silicon substrate. The ungrafted polymer was washed with toluene to remove excess materials, obtaining a monolayer PS brush on the substrate. PS polymer brushes are generally considered to be the best form for surface treatment because of the formed strong covalent bonds between the substrate and the end of PS.^29,30^ It has better molecular interactions with the PS matrix, which contains hexagonal PMMA cylinders. By simultaneously controlling the interfacial interactions and the thickness of the BCP film, we can dictate the desired orientation. There are many hexagonally packed PMMA cylinders within the PS matrix, as shown in Fig. 1(b). Thereafter, the PS-*b*-PMMA2 solution was spin-coated onto the silicon substrate treated by the PS–OH brush, and the annealing was performed at a higher temperature than the glass transition temperature of BCPs in optimized vacuum thermal annealing to induce the microphase separation.

**Fig. 2:**
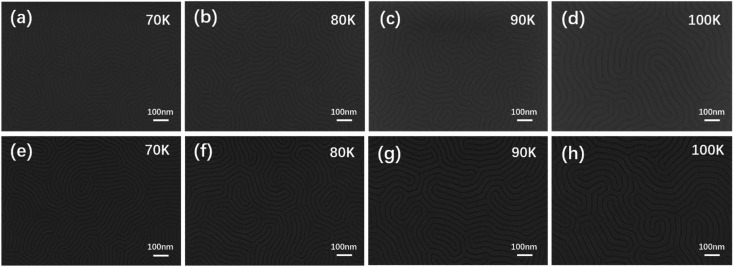
SEM images of PS-*b*-PMMA1 film annealed under the same condition. The PS-*b*-PMMA1 microphase separation morphologies under an optional annealing temperature condition at different magnifications without dry etching: (a) 70 K; (b) 80 K; (c) 90 K; and (d) 100 K; and with dry etching: (e) 70 K; (f) 80 K; (g) 90 K; and (h) 100 K on the four same substrates. The thickness of all films is about 32.6 nm.

The balanced interface interaction of BCP with its corresponding substrate surface and the free surface can induce the microphase domains for perpendicular or parallel orientation, making the BCP self-assembly films as pattern templates for nanowire array fabrication in future advanced semiconductor devices. Fig. 2 shows the SEM images of the optimal PS-*b*-PMMA1 thin films by vacuum thermal annealing at 240 °C for 4 minutes. They demonstrate perfect self-assembly microphase separation patterns. Fig. 2(a)–(d) show fingerprint patterns without defects on four substrates with different SEM magnifications, respectively, indicating that the vacuum annealing conditions in these samples were optimized, effective, stable, and repeatable to achieve microphase separation of PS-*b*-PMMA1. Obviously, under a suitable high-temperature condition, the vacuum annealing time can be shortened to make PS-*b*-PMMA1 microphase separation more efficient and sufficient. Meanwhile, it should be considered that the high-temperature annealing time has an upper limit to ensure the uniformity of the BCP film and that the film will not be damaged. Considering the defect ratio and high resolution to match the film thickness, the best annealing process condition to achieve self-assembly is vacuum annealing at 240 °C for 4 minutes after a set of experiments. In the case of two different BCP domains, such as lamella or cylinder phase, controlling the lamellar microdomains to align perpendicular to the sidewalls of silicon substrate trench edges or cylindrical microdomains to be parallel along the sidewalls of silicon substrate trench edges is much essential for the generation of DSA nanopatterns. Fig. 2(e)–(h) demonstrate the fingerprint patterns with high contrast without defects on four substrates (corresponding to Fig. 2(a)–(d)) after the same etching condition under different SEM magnifications.

**Fig. 3:**
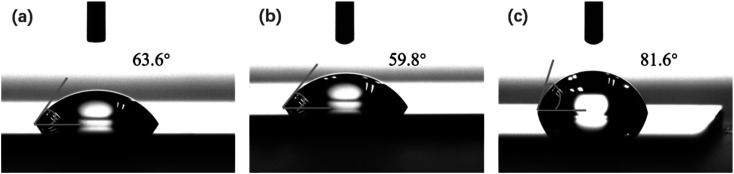
Water contact angle of Si substrate (a) before cleaning, (b) after Si substrate cleaning, and (c) after PS–OH brush grafting.

Meanwhile, for the BCP cylindrical microdomain self-assembly of PS-*b*-PMMA2, we also achieved good self-assembly patterns without any etching conditions on the same cleaned substrate. When the precise film thickness is not controlled, the PMMA cylinders in the PS matrix micro-domains cannot be seen under high-power SEM. When we accurately control the film thickness, the PMMA cylinders in the PS matrix micro-domains will be easily observed using a high-power SEM. We have overcome the process difficulties and realized the microphase separation morphology of parallel cylinder nanowires without any gas etching, thus reducing process steps and costs for subsequent device-related research. Certainly, some cleaning steps are required during the microphase separation of PS-*b*-PMMA2. If the substrate is not cleaned by the wet method before PS-*b*-PMMA2 spin coating, the microphase separation cannot be observed after vacuum thermal annealing. Because the PS–OH brush contains hydroxyl groups at the end, it can dehydrate with the hydroxyl groups on a cleaned silicon substrate or a silicon substrate etched by oxygen plasma, thus grafting on the substrate surface.^29,30^ In our experiments, before and after the acetone and isopropanol (IPA) cleaning process at 33 °C, the surface water contact angle of the substrate was measured as 63.6° and 59.8°, respectively, as shown in Fig. 3(a) and (b). The substrate surface tends to be hydrophilic due to the removal of organic contaminants on the substrate.^31–33^ After spin-coating PS–OH, subsequent annealing, and then cleaning with toluene, we tested the water contact angle of the surface grafted with the PS–OH brush, which was 81.6° with a hydrophobic character, as shown in Fig. 3(c).

**Fig. 4:**
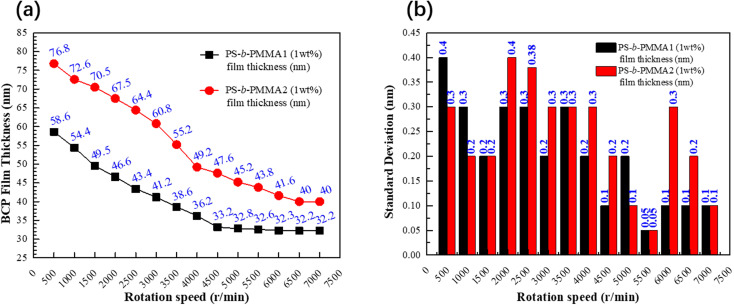
(a) Statistical analysis of the film thickness with different rotation speeds of PS-*b*-PMMA1 and PS-*b*-PMMA2 at the same concentration of 1 wt%. (b) Distribution of standard deviations for 10 samples along each film thickness corresponding to the rotation speeds. All the standard deviations are in the range of 0.05–0.4 nm.

Certainly, the regulation of the BCP film thickness was also very important. PS-*b*-PMMA1 and PS-*b*-PMMA2 with the same 1 wt% concentration were spin-coated onto the corresponding respective modified substrates. The corresponding relationship between the film thickness and different rotation speeds of PS-*b*-PMMA1 and PS-*b*-PMMA2 with the same mass fraction is shown in Fig. 4(a). In this process, each spin coating experiment PS-*b*-PMMA1 and PS-*b*-PMMA2 was carried out five times respectively. To further verify the stability of film thickness, we calculated and analyzed the population standard deviation of these data. The population standard deviation (SD) can be estimated as follows:1
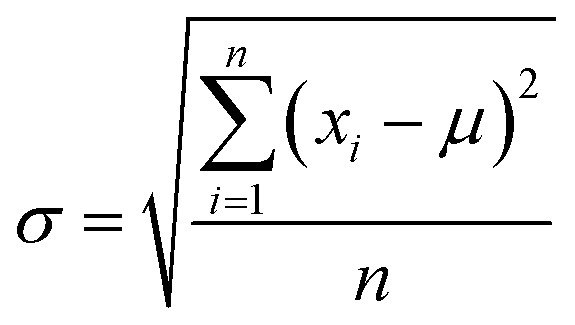
where *σ* is the SD value, *i* and *n* are integers, *x*_*i*_ is the number of BCP film thickness measurements at the same number of revolutions (nm), and *μ* is the weighted sum average of *x*_*i*_ (nm). The SD values of block copolymers PS-*b*-PMMA1 and PS-*b*-PMMA2 are shown in Fig. 4(b). The amplitude of the SD corresponding to each test point is small, which shows that the robustness of the accurate film thickness is very good, and the measurement accuracy is also very high.

**Fig. 5:**
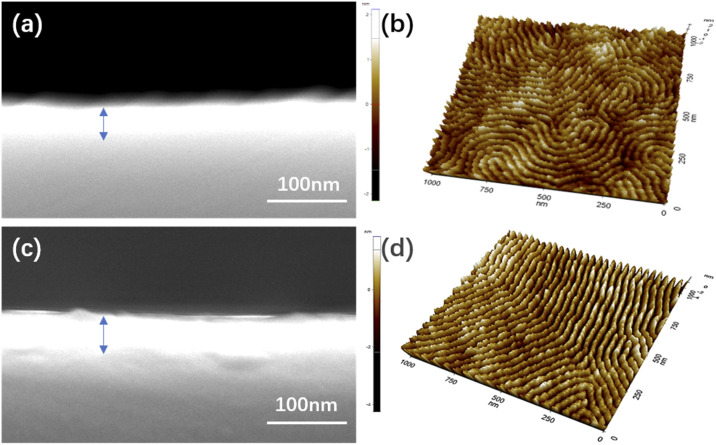
SEM images of the film thickness of (a) PS-*b*-PMMA1 and (c) PS-*b*-PMMA2 after thermal annealing, respectively. AFM three-dimensional topographies of (b) PS-*b*-PMMA1 and (d) PS-*b*-PMMA2 films after etching, respectively.

Both BCPs show different film thicknesses at the same speed. When the spin coating speed of both BCPs reaches a certain speed, the film thickness remains constant with the increase in spin-coating speed. In particular, the commensurability between the pitch of block copolymers and the film thickness is a crucial influence factor in achieving the well-ordered self-assembled morphology.^34,35^ In this article, the optimal film thicknesses of PS-*b*-PMMA1 and PS-*b*-PMMA2 from Fig. 4(a) and (b) are 32.6 nm and 43.8 nm, as shown in Fig. 5(a) and (c), respectively, because the film thickness and the corresponding self-assembly pitch ratio of the two kinds of BCP are almost 1 : 1, which has the characteristics of one-pitch film.^36–38^ Meanwhile, to improve the contrast between the PS block and the PMMA block in PS-*b*-PMMA1 and to remove the surface PS block layer because of the PS-wrapped PMMA block in PS-*b*-PMMA2, oxygen plasma etching on both BCP self-assembly microphase separation morphologies was performed for 5 s. We further scanned it with AFM, and the three-dimensional morphology also shows its self-assembly morphology, as shown in Fig. 5(b) and (d). Indeed, controlling the BCP thin film domain orientation and alignment is essential for future applications related to semiconductors and so on.^39–41^

**Fig. 6:**
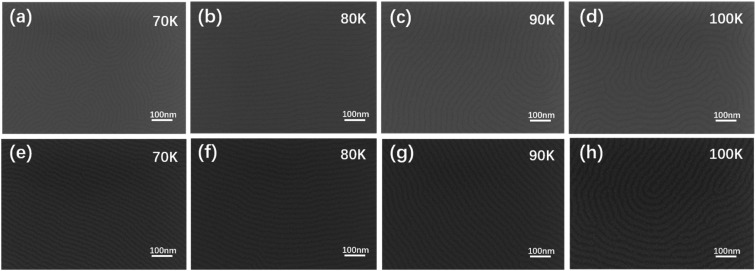
SEM images of the PS-*b*-PMMA2 film annealed under the same condition. The PS-*b*-PMMA2 microphase separation morphologies under an optional annealing temperature condition at different magnifications without dry etching: (a) 70 K; (b) 80 K; (c) 90 K; and (d) 100 K; and with dry etching: (e) 70 K; (f) 80 K; (g) 90 K; and (h) 100 K on the four same substrates. The thickness of all films is about 43.8 nm.

To balance and optimize the defectivity and productivity of the process, several experimental studies were carried out to find the best thermal annealing process window. For PS-*b*-PMMA2 on the substrate with a PS–OH brush, a series of experiments including various annealing times under the conditions of various thermal annealing temperatures were conducted, and an optimal condition (240 °C/4 min) was obtained. The SEM images demonstrated a defect-free cylindrical microdomain pattern with four repeating experiments, as shown in Fig. 6(a)–(d). The domain orientation relative to the Si substrate is mainly controlled by the interaction of each block with the substrate and the free surface *via* polymer confinement and wetting energetics, including commensurability effects that result from the matching or mismatching of the film thickness with the pitch of PS-*b*-PMMA2. The SEM images demonstrate microphase separation morphologies without defects, indicating the self-assembly pattern of parallel PMMA cylinders in the PS matrix without any etching being achieved. It has been reported that surface treatment has a positive impact on the self-assembled morphologies and repeatability, which promotes the parallel orientation of cylindrical domains. Because of incompatibility and the repulsion forces between the two blocks in the diblock copolymer, PS-*b*-PMMA2 was segregated into microdomains to achieve minimal free energy, forming a stable state after the thermal-driven microphase separation process. Those ordered arrangements indicate that the system has an equilibrium state, that is to say, the free energy of the system in the appropriate annealing process window is lower than that of the disordered state. For the PS-*b*-PMMA2, the PMMA block (dark grey) and PS block (light) represent the two polymer blocks with different chemical properties. Similarly, Fig. 6(e)–(h) demonstrate a defect-free cylindrical microdomain pattern on the same four substrates after the etching condition at different SEM magnifications, corresponding to Fig. 6(a)–(d) respectively.

**Fig. 7:**
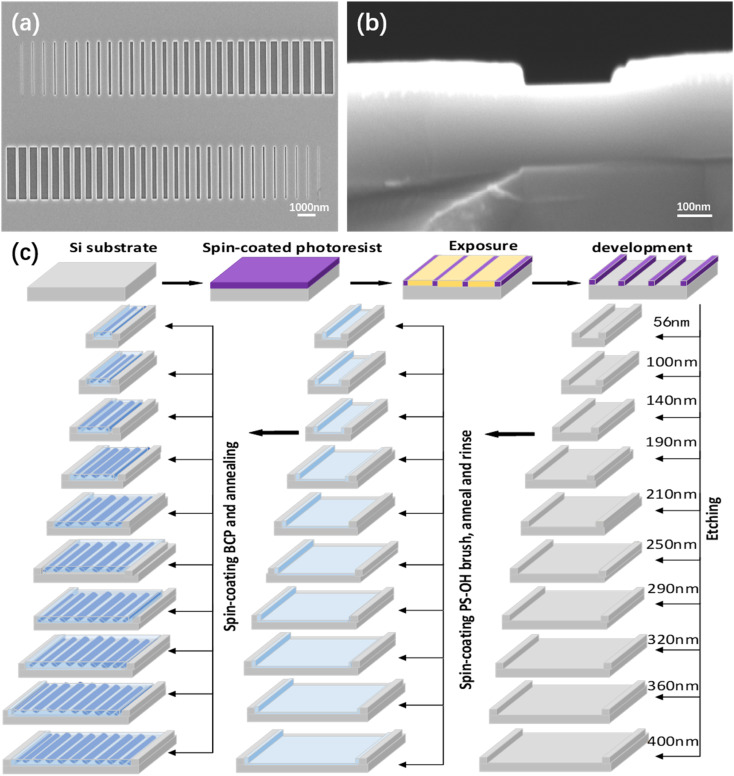
(a) SEM images of groove substrates with different sizes. (b) SEM image of the trench with a width of 280 nm and a trench sidewall height of 55 nm. (c) Schematic of the DSA process of PS-*b*-PMMA2 by a graphoepitaxy strategy.

**Fig. 8:**
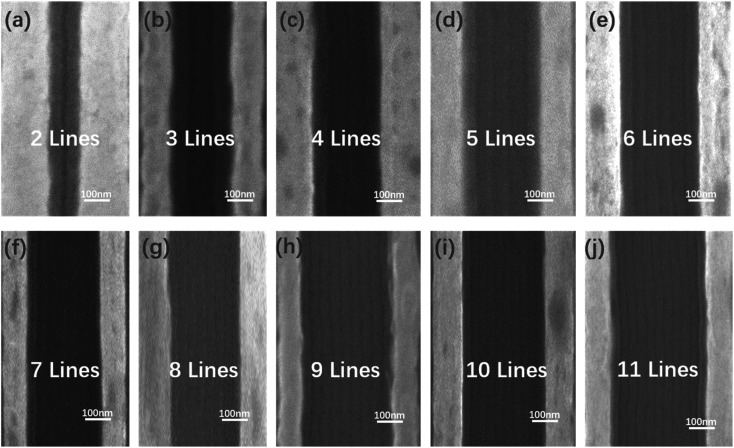
SEM images of the PS-*b*-PMMA2 directed self-assembly in the topographic confinement with (a) 56 nm, (b) 100 nm, (c) 140 nm, (d) 190 nm, (e) 210 nm, (f) 250 nm, (g) 290 nm, (h) 320 nm, (i) 360 nm, and (j) 400 nm width and 55 nm depth. Different parallel cylinder phases can be seen in the different manufactured trenches, and the bright lines are sidewall edges of these trenches.

Generally, the molecular chains of kinetically frozen cast films can get enough energy from thermal annealing to promote the chain motion and microphase separation of BCP, achieving ordered self-assembly morphology. In this way, the BCP will achieve an equilibrium structure. It was observed that lamellar microdomains of PS-*b*-PMMA1 can still form good quality fingerprint patterns with a pitch size of about 32 nm. Meanwhile, parallel-oriented cylindrical microdomains of PS-*b*-PMMA2 can be achieved in good quality fingerprint pattern with a pitch size of about 38 nm, as the grafted polymer brush has been used to tune the wettability of the substrate surface for their energy corresponding to the BCP microphase separation. The optimum use of the polymer brush layer provides composition-dependent interfacial energy tunability, resulting in defect-reduced SA and DSA of BCP nanostructures. This means that vertically orientated lamella-forming nanostructures perpendicular to trench edges for PS-*b*-PMMA1 and parallelly orientated cylinder-forming nanostructures along trench edges for PS-*b*-PMMA2 can both be formed using a specific range of interfacial energy-matched polymer brushes and BCP thicknesses. Defect-free SA and DSA of PS-*b*-PMMA1 (or PS-*b*-PMMA2) can also be achieved by precisely designing and fabricating the trench width and depth that are compatible with the microphase separation period of the BCP. DSA of BCP allows us to implement precise directional alignment by employing graphoepitaxy guiding constraints, which is one of the most important ways to realize integration for future devices. The schematic diagram in Fig. 7 shows the DSA process of PS-*b*-PMMA2 with graphoepitaxy. In this process, the grooved substrate was finely manufactured by EBL. The graphoepitaxy approach is favorable for forming nano features that are strict registration and parallel alignment with the sidewall. Nano lines with large area continuity and alignment are suitable for evaluating the characteristics of devices such as nano line gas sensors and Schottky source/drain multi-nanowire-channel devices.^42,43^ It should be noted that the three microns correlation length of cylindrical microdomains is one of the key parameters. The 55 nm-deep trench templates with different widths (a: 56 nm; b: 100 nm; c: 140 nm; d: 190 nm; e: 210 nm; f: 250 nm and so on) were fabricated by EBL, and the substrate and sidewalls of the groove would be functionalized with the PS–OH brush and then coated with PS-*b*-PMMA2. Fig. 7(a) shows the prepared grooves of different widths and the same depth. The SEM image of the groove with a width of 250 nm and sidewall height of 55 nm is presented in Fig. 7(b). Fig. 7(c) depicts the schematic diagram of different graphoepitaxy processes of PS-*b*-PMMA2 with different numbers of PMMA cylinders parallel to the corresponding trench sidewalls. From the molecular characteristics of microphase separation of the diblock copolymer, the cylindrical PMMA microdomain was assembled into the PS matrix by post-optimized thermal annealing. Some cylinders formed within a limited groove substrate width, *W*, of the wetting condition are symmetric, in which *W* (*nL*_0_) is physical trench width minus the PS–OH brush layer thickness on both substrate sidewalls attractive to PS. The number of PMMA cylinders was two lines in the 56 nm trench, three lines in the 100 nm trench, four lines in the 140 nm trench, five lines in the 190 nm trench, six lines in the 210 nm trench, seven lines in the 250 nm trench, eight lines in the 290 nm trench, nine lines in the 320 nm trench, ten lines in the 360 nm trench and eleven lines in the 400 nm trench, respectively, as shown in Fig. 8. It is noted that the width or pitch of cylinders at the center of the trench is larger than those located at trench edges. It may be caused by thickness variations due to capillary effects because the trench deeper is bigger than the PS-*b*-PMMA2 film thickness, or the uneven distribution of the sidewall force by the sidewall brushes, and the middle BCPs have large freedom to self-assemble. Especially, when the groove width is fairly small, the cylindrical domains' parallel orientation of PS-*b*-PMMA2 is greatly affected by the sidewall and the grafted PS–OH brush. When the grooves with a suitable width are applied, the most central parallel orientation of cylindrical nanowires forms a compatible micro-phase separation pitch with the same self-assembly pitch on the bare substrate. These topographic features enhance the self-assembled micro-domain order of PS-*b*-PMMA2 along the trench sidewalls and limit the dewetting caused by macromolecule diffusion during the thermal annealing process.

**Fig. 9:**
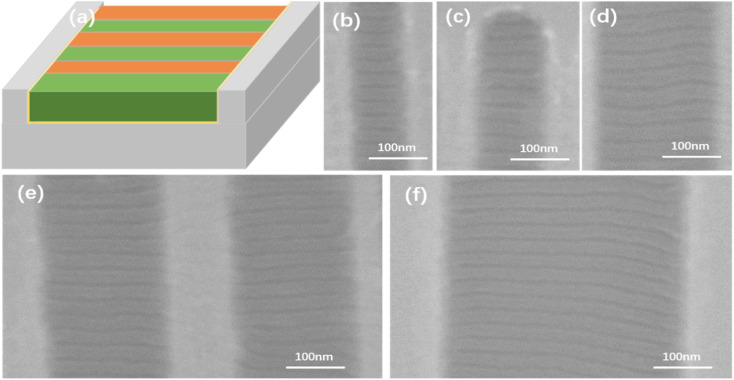
(a) Schematic illustration of the directed assembly of PS-*b*-PMMA1, that is the lamellar phase of the PS-*b*-PMMA1 is perpendicular to both trench bottoms and sidewalls. SEM images of PS-*b*-PMMA1 with the lamellar phase align perpendicular to the trench sidewall edges in the topographic confinement with (b) 56 nm, (c) 100 nm, (d) 190 nm, (e) 250 nm, and (f) 360 nm width and 55 nm depth. The self-aligned pattern where the trench bottom and sidewalls are neutral to the lamellar phase PS-*b*-PMMA1 diblock copolymer. Perpendicular lamellae can be seen in the different trenches, and the bright lines are the edges of these trench sidewalls.

Furthermore, we also modified the bottom and sidewalls of the trenches with a neutral random copolymer brush corresponding to the lamellar PS-*b*-PMMA1. The neutral trench bottom is wet equally to PS and PMMA blocks, so that the PS-*b*-PMMA1 material within the trench orients perpendicularly to the Si substrate surface. Meanwhile, the neutral trench sidewalls promote equal PS and PMMA wetting, resulting in alternating lamellar domains spanning the trench width and running orthogonal to the trench sidewalls. The microphase separation pitch of PS-*b*-PMMA1 is 32 nm. Hence, by combining nonpreferential brush layers with the same groove template, the directed self-assembly of perpendicular lamellae of PS-*b*-PMMA1 was successfully achieved. Particularly, under the circumstance of lamellar PS-*b*-PMMA1, it is desirable to achieve lamellar microdomains align perpendicular to the sidewalls of trench edges, as it may be valuable for future nanowire array devices.^44^ Both the sidewalls and trench bottoms have been modified with a nonpreferential PS-*r*-PMMA–HEMA brush to induce the perpendicular lamellar domains that align perpendicular to the trench sidewalls, which is the critical process, as shown in Fig. 9(a). Different-sized silicon grooves neutralized by the PS-*r*-PMMA–HEMA brush were fabricated for DSA of PS-*b*-PMMA1, and the SEM images of the experimental results are shown in Fig. 9(b)–(f). We have focused on the topographic confinement width and the film thickness dependence of the microdomain orientation because of the interfacial interaction of PS-*b*-PMMA1 with the trench sidewall and the trench bottom. Microdomain orientation depends on the BCP film thickness when the interfacial interaction between the trench substrate and each block in the BCP is equal to the nonpreferential wetting conditions. Alignment of lamellae perpendicular to the sidewalls of trench edges *via* PS-*r*-PMMA–HEMA brush modification of the topographic features is the result of our experimental analysis. It has been reported that without the homogeneous PS-*r*-PMMA–HEMA brush modification, people could not realize this feature at all.^45^ In other words, on PS-*r*-PMMA–HEMA surfaces, lamellar domains of the PS-*b*-PMMA1 are oriented perpendicular to both the trench sidewalls and the trench bottom. In this paper, we go through a series of process conditions by adjusting the surface energy of the trench sidewalls and the trench bottom surface to be nonselective, especially in the substrate grooves, (b) 56 nm, (c) 100 nm, (d) 190 nm, (e) 250 nm, (f) 360 nm width and 55 nm depth, in which the alignment of lamellae perpendicular to the trench sidewalls is also demonstrated. Indeed, it is sometimes affected by the large roughness of the groove sidewall. An intentionally matched edge of the pinning pattern with the trench guiding patterns was to emphasize the effect of the border on pinning lamellar microdomain alignment of PS-*b*-PMMA1 lamellar microdomains. The approach removes the sidewall roughness and undesirable reflection in the lamellar nanowire edge roughness. Both PS and PMMA in PS-*b*-PMMA1 provide an energetic mechanism for the lamellar phase precise registration, in which experimental results and correlation analysis indicate the critical role of the nonpreferential wetting conditions and the pinning pattern for the alignment and registration.

**Fig. 10:**
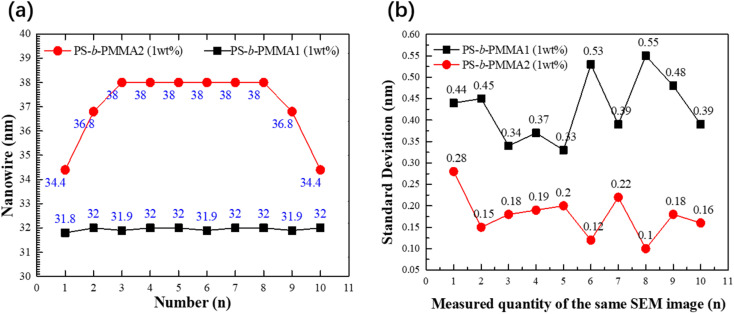
(a) Nanowire width variation of directed self-assembly of block copolymers PS-*b*-PMMA2 and PS-*b*-PMMA1 in a 360 nm grooved substrate. (b) Distribution of standard deviations for 10 samples along each measurement in the same SEM image. All the standard deviations are in the range of 0.1–0.55 nm.

The nanowire pitch width of the directed self-assembly morphology of PS-*b*-PMMA2 and PS-*b*-PMMA1, as shown in Fig. 10(a), can be obtained from the high-resolution SEM images in Fig. 8(i) and 9(f) of the DSA of BCP in the same constraint groove with 360 nm width. The experiments demonstrate the same results for the other 10 groups under the same experimental conditions, and SD was also calculated, as shown in Fig. 10(b). The amplitude of the SD corresponding to each test group, is small, indicating that the accuracy and quality of nanowire width measurement of both PS-*b*-PMMA2 and PS-*b*-PMMA1 are very high. A gradient change in the linewidth from the groove edge to the center is observed, because of the possible interface interaction between the blocks and the confining sidewall as discussed above. When the parallel orientation of cylindrical domains of these experiments has an even number of lines in the substrate groove and the lines are close to the centerline, it appears to be a gradual phenomenon. The middle region of the nanowire achieves precisely the same period as its self-assembly, while the lines located at two sides gradually decrease. It shows that the surface chemistry of the confining sidewall will affect the pattern formation in several periods, and this effect will weaken with the increase in distance, as shown for PS-*b*-PMMA2. However, for PS-*b*-PMMA1, the width of their nanowires does not change significantly with the rise in the number of measurements and is almost as large as its pitch. This is because of the interaction between the polymer molecules and the molecular dynamics of the uniform neutralization brush on the sidewall of the substrate, for lamellar PS-*b*-PMMA1 in a neutral brush-modified groove.

The above-mentioned results and analysis show the relevant details of the parallel orientation of the cylindrical microdomains of PS-*b*-PMMA2 and the lamellar microdomains PS-*b*-PMMA1 aligned perpendicular to the trench sidewall edges. For the PS-*b*-PMMA2 cylindrical microdomains, the center nano lines match their period, and the nano lines at the edge are compressed into a non-uniform structure. It may be due to the different affinities between the confining sidewall modified by the PS–OH brush and BCP located at different distances from sidewalls. Balancing the interfacial interactions of PS-*b*-PMMA2 with the confining substrate groove and its free surface could induce the cylindrical domains' parallel orientation, permitting the films to serve as templates or nanowire array gas sensor devices in future nanofabrication. For the PS-*b*-PMMA1 lamellar microdomains, the lamellar domains are directed perpendicular to both sidewalls and the trench bottom. Meanwhile, the sidewalls of the trenches with a neutral random copolymer brush show the same affinity to PS and PMMA blocks, so that the BCP material within the trench orients perpendicularly to the substrate surface. In addition, the neutral trench sidewalls also promote equal PS and PMMA wetting, resulting in alternating lamellar domains spanning the trench width and the lamellar microdomains perpendicular to the trench long axis to minimize the energy of the system. Particularly, in the grooves with 56 nm, 100 nm, 190 nm, 250 nm, and 360 nm widths and the same 55 nm depth, for PS-*b*-PMMA1 and PS-*b*-PMMA2, it not only realized the DSA of cylindrical domain parallel orientation along sidewalls but also realized the DSA of the lamellar microdomains perpendicular to both trench sidewalls and the trench bottom with the same process and under the annealing conditions using different types of BCP and the corresponding brushes. Simultaneously, this work provides essential experimental process conditions, theoretical analysis, and valuable insights for DSA lithography to form nanopatterning in future state-of-the-art semiconductor devices.

## Conclusion

In summary, this work focused on exploring the self-assembly and directed self-assembly morphologies of both lamellar PS-*b*-PMMA1 and cylindrical PS-*b*-PMMA2 on their respective brush-modified Si substrate under variable process conditions, which may offer some valuable reference for future applications in semiconductor device pattern transfer technology. The concentration of the BCP, cleaning of the substrate, grafting of the neutral brush on the Si substrate, the thickness of the BCP film, annealing temperatures and durations, and manufacturing of substrate grooves with different widths of 56 nm, 100 nm, 190 nm, 250 nm, 360 nm, and the same 55 nm depth were applied to explore their influences on SA and DSA of two different types of BCPs. With the same substrate grooves modified by different brushes, different morphologies were achieved by the DSA *via* the graphoepitaxy method. The mechanism of both the orientation of cylindrical domains parallel along trench sidewalls and the orientation of lamellar microdomains perpendicular to trench sidewalls and the trench bottom has been deeply analyzed. Achieving low defect density and high productivity from self-assembly to directed self-assembly of both PS-*b*-PMMA1 and PS-*b*-PMMA2 under optimal process conditions also provided valuable reference information. These combined conditions should be accurately controlled to ensure minimal performance changes to achieve large-scale manufacture in future integrated circuit semiconductor devices.

## Conflicts of interest

The authors declare no competing financial interest.

## Supplementary Material
